# Peripheral Parenteral Nutrition and Activities of Daily Living in Hospitalized Older Frail Patients

**DOI:** 10.7759/cureus.44423

**Published:** 2023-08-31

**Authors:** Shunsuke Soma, Yuuichi Tazawa, Shin Yamada, Nao Szuki, Daiki Narita

**Affiliations:** 1 Emergency and Critical Care Center, Aomori Prefectural Central Hospital, Aomori, JPN; 2 Nutrition Management, Aomori Prefectural Central Hospital, Aomori, JPN; 3 Rehabilitation, Aomori Prefectural Central Hospital, Aomori, JPN

**Keywords:** hospitalization, parenteral nutrition, activities of daily living, frailty, frail elderly

## Abstract

Background: Frail older adults require nursing care following hospitalization for acute illnesses. Frailty is reversible, and appropriate nutritional management and rehabilitation during hospitalization are essential. However, optimal nutritional management for patients who are unable to obtain adequate nutrition via oral intake has not been established. We aimed to determine whether peripheral parenteral nutrition (PPN) promotes the recovery of activities of daily living (ADLs) in frail older patients.

Methods: This was a retrospective, observational cohort study conducted at the General Medicine Department of Aomori Prefectural Central Hospital in Aomori, Japan. The primary outcome was recovery of the Barthel index (BI) from the beginning of rehabilitation to discharge, and the secondary outcomes were the proportion of patients transferred for rehabilitation and the nutritional status.

Results: In total, 342 patients hospitalized during the period of April 2018 to January 2022 were included, of whom 127 (37.1%) received PPN and 215 (62.9%) did not. Contrary to our expectations, recovery of the BI was lower in the PPN group than that in the non-PPN group (12.2 (95% confidence interval (CI): 8.5-16.0) vs. 22.4 (18.8-23.0); p < 0.01). Multivariable analysis revealed PPN as an independent risk factor for poor BI recovery (mean difference = -7.3 (95% CI = -12.7 to -1.9)).

Conclusion: Nutritional management through PPN for frail older adults may not improve physical activity. The nutritional management of frail patients with inadequate oral intake remains challenging.

## Introduction

Frailty is a state of decreased reserve capacity and stress recovery with age. The prevalence of frailty among community-dwelling older adults is reportedly associated with age [1]. As the proportion of older adults in Japan has increased dramatically over the past few decades, the prevalence of frailty is also expected to increase [2]. Frailty is strongly associated with negative health outcomes, such as mortality, hospitalization, a decline in activities of daily living (ADLs), and emergency department visits [3]. Frail patients can easily experience a decline in ADLs and impair their daily life when they sustain even minor injuries, such as falls or mild infections. Even if the acute illness that triggers the condition is not serious and the patient is quickly cured, recovery of their ADLs to pre-admission conditions takes time, and they often cannot return home. Meanwhile, frailty is reversible and can be improved with nutritional intervention, exercise therapy, vitamin D supplementation, and a reduction in polypharmacy [4]. Therefore, management of frail older adults during hospitalization needs to be understood.

Numerous interventional studies have been conducted in frail older adults. Regarding interventions, a network meta-analysis revealed that exercise therapy was the most effective in reducing frailty, followed by a combination of exercise and nutritional therapy [5]. Nutritional therapy alone is often insufficient [6]. However, frailty and malnutrition are strongly associated [7,8], and appropriate nutritional management in frail older adults is likely to improve muscle mass, strength, and physical activity. Nutritional therapy reportedly contributes to improvements in frailty indices and physical activity, especially in malnourished older adults, when combined with other interventions, especially exercise therapy [9,10]. However, in many cases, the necessary energy cannot be obtained via the oral route. Frail patients admitted to hospitals with acute illnesses often do not have an adequate oral intake, even with oral nutritional supplements, because of a decreased appetite. In such cases, peripheral parenteral nutrition (PPN) has been widely used because peripheral venous tracts are easier to puncture than central venous tracts and are more easily combined with oral intake than nasogastric tube feeding [11]. However, we are not aware of reports that nutritional supplementation through PPN improves the nutritional status and promotes the recovery of physical activity in frail older patients. Therefore, we conducted this study, aiming to investigate whether nutritional management using PPN is useful in restoring ADLs of frail older patients who are hospitalized for acute illness and unable to obtain adequate nutrition via oral intake.

## Materials and methods

Study design, setting, and participants

This was a retrospective cohort study conducted on frail older patients admitted to the General Medicine Department of Aomori Prefectural Central Hospital in Aomori, Japan, from April 1, 2018 to March 31, 2022. Aomori Prefectural Central Hospital is a tertiary acute care hospital, and most inpatients in the General Medicine Department are admitted from the emergency department. The inclusion criteria were being aged 65 years and older, hospitalized for at least one week, meeting frailty criteria (Clinical Frailty Scale (CFS) score >3), not obtaining enough energy from the diet (taking in less than half of the food provided in the first week of admission), and being in need of rehabilitation as determined by the attending physician. The exclusion criteria were being hospitalized for less than one week, being bedridden with full ADL assistance (CFS score = 8) or considered in the terminal stage at the time of admission (CFS score = 9), and undergoing only eating and swallowing rehabilitation. This study was approved by the Ethics Committee of Aomori Prefectural Central Hospital (approval number: R03-2-064). A summary of the study was posted on the hospital’s website, and information was provided about how patients could opt out, which allowed eligible patients to be removed from the registry at any time.

Frailty assessment

The most widely used frailty criteria are the Cardiovascular Health Study (CHS) criteria, which have been revised to meet the Japanese criteria (J-CHS criteria) [12]. However, these criteria require the measurement of grip strength and gait speed. Although extremely useful for the identification of frail older adults in the community-dwelling population, it is inappropriate for frail patients who are hospitalized for acute illness, as they often have substantially impaired physical function and mobility. One needs to estimate whether the patient was frail before they became acutely ill. The most accurate and reproducible way to assess a patient’s condition before hospitalization is to ask their family or caregivers about their prehospitalization ADLs. Therefore, in this study, we employed the CFS, which estimates frailty based on a patient’s ADLs [13]. Prehospitalization ADLs were assessed by integrating information from the nursing care record incorporated into the Japanese long-term care insurance system, information from the initial emergency medicine residents who performed the life history interview, information from the nurse present at the time of admission, and information from the physical therapists present at the start of the patient’s rehabilitation.

Data collection and outcomes

Almost all of the main information was gathered from medical records from March 8 to May 9, 2022. Additional data, including the patients’ primary and secondary illnesses, intensive care unit (ICU) or high care unit (HCU) stay, rehabilitation time, and percentage of sufficient oral energy intake, were collected during March and April 2023. The study was not blinded and the author/analyst had access to the participants’ personally identifiable information during the data collection period. Almost all information, including patient age, sex, dietary intake, nutritional assessment, length of hospital stay, patient transferal status, reason for discharge, complications of catheter-related blood stream infection, use of tube feeding, and use of central venous nutrition, were extracted from the electronic medical records. The Barthel index (BI) from admission to discharge and the total rehabilitation time during hospitalization were obtained from a database independently managed by the rehabilitation department.

The primary factor was whether PPN was used, and patients were included in the PPN group if PPN was used at least once during the hospital stay. PPN comprised a preparation containing amino acids, glucose, and vitamin B1, corresponding to Bfluid (Otsuka Pharmaceutical Factory, Inc., Tokyo, Japan) and Pareplus (Yoshindo Inc., Toyama, Japan). Patients receiving fat emulsions alone were not included in the PPN group. The primary outcome was the difference in the BI between the beginning and end of rehabilitation. Secondary outcomes included the percentage of patients transferred for rehabilitation and serum biochemical nutritional indices (total protein, albumin, urea nitrogen concentration, and total lymphocyte count) between admission and discharge. The safety indices were mortality and the prevalence of peripheral venous catheter-related bloodstream infections.

Statistical analysis

Continuous variables that were normally distributed were expressed as means ± standard deviations, and the difference between the two groups was tested using an unpaired t-test. Non-normally distributed variables were expressed as medians and quartiles, and the difference between the two groups was tested using the Mann-Whitney U test. Categorical variables were expressed as proportions and the difference between the two groups was tested with the χ2 test, or the Fisher exact test if the categorical variable size was less than 20. In addition, multiple regression analysis was performed to determine whether PPN use independently affected changes in the BI. The following variables were considered relevant based on previous studies [14-20] and discussions with several physical therapists on our team: age, concomitant tube feeding, concomitant central venous nutrition, dementia, BI at the beginning of rehabilitation, serum albumin concentration at admission, ICU or HCU stay, and Charlson Comorbidity Index (CCI). Stratified analysis was performed according to whether the patient had a primary or secondary gastrointestinal disease, time spent in rehabilitation, and proportion of energy intake relative to basal energy expenditure. Sensitivity analysis was performed on the sample by including patients who were discharged because of death and assigning them a BI of 0. As less than 5% of the variables required for the analysis of the primary outcome were missing, data with missing values were not analyzed. Statistical significance was set at p < 0.05, and all tests were two-tailed. Statistical analyses were performed using Stata version 17 software (StataCorp LLC, College Station, TX, USA).

## Results

**Figure 1:**
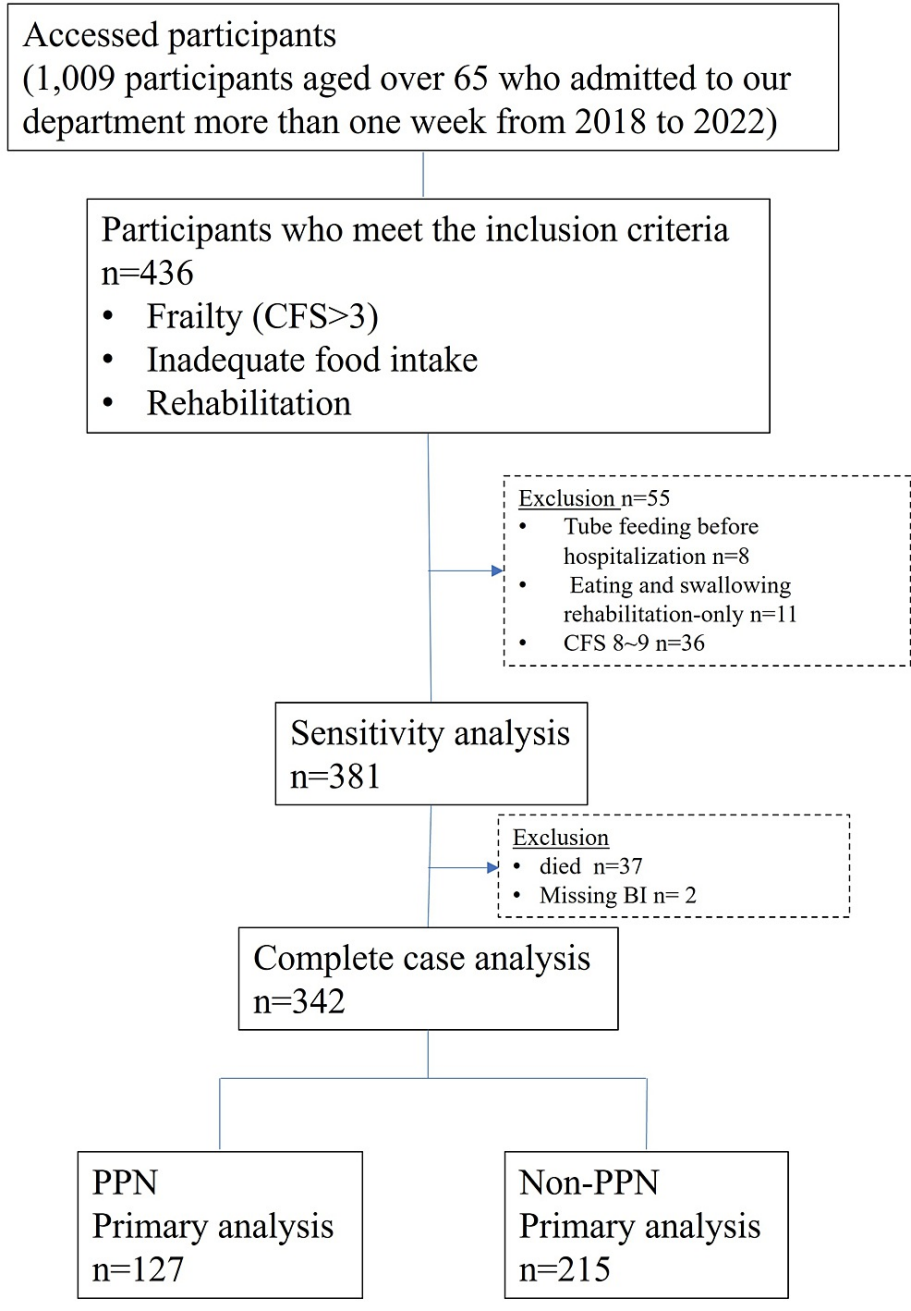
Enrollment, exclusion, and follow-up CFS, clinical frailty scale; BI, Barthel index; PPN, peripheral parenteral nutrition

Of the patients admitted to our General Medicine Department from April 1, 2018 to January 31, 2022, 1,009 were aged ≥65 years and had been hospitalized for more than a week. Of these, 436 met the inclusion criteria of having a CSF score >3, eating less than half the food provided during the first week of admission, and being rehabilitated during the hospital stay. Of these, eight patients who were tube-fed before admission, 11 who underwent only feeding and swallowing rehabilitation, and 36 who had CSF scores of 8-9 or were considered terminal at the time of admission were excluded. Complete case analysis was performed on 342 patients after excluding 37 who died during hospitalization and two for whom the BI was missing (Figure 1).

Table 1 summarizes the patient background characteristics. The PPN group had a longer hospital stay and a higher percentage of patients being tube-fed than the non-PPN group. The median (interquartile range) BI at the start of rehabilitation was significantly lower in the PPN group than that in the non-PPN group (0 {0-5} vs. 0 {0-10}). However, the BI change did not appear to be clinically meaningful. Similarly, serum albumin concentrations at admission were significantly lower in the PPN group than those in the non-PPN group (mean ± standard deviation: 3.1 ± 0.6 vs. 3.2 ± 0.7 mg/dL), but the difference was small and not clinically meaningful. Disease categories in the PPN and non-PPN groups were not uniformly distributed, but factors affecting outcomes, such as age, sex, presence of dementia, CCI, and nutritional indicators at admission, did not significantly differ between the two groups.

**Table 1:** Characterization of the participants according to the usage of peripheral parenteral nutrition PPN, peripheral parenteral nutrition; ICU, intensive care unit; HCU, high care unit; BI, Barthel index; MNA-SF, Mini Nutritional Assessment Short-Form; TP, total protein; ALB, albumin; BUN, blood urea nitrogen; TLC, total lymphocyte count; BEE, basal energy expenditure

			Total (n=381)	PPN (n=144)	Non-PPN (n=237)	p-value
Age, year			82.9 (7.7)	82.5 (7.8)	83.7 (7.6)	0.129
Gender, *n*	Male		158 (41.5)	65 (45.1)	93 (39.2)	0.257
	Female		223 (58.5)	79 (54.9)	144 (60.8)	
Hospitalization period, days			24 (15-34)	28 (19-38)	22 (15-30)	0.000
ICU or HCU stay, n			117 (30.7)	37 (25.7)	80 (33.8)	0.098
Disease category, *n*	Respiratory		164 (43.0)	72 (50.0)	92 (38.8)	0.000
	Nephrological		50 (13,1)	12 (8.3)	38 (16.0)	
	Infection		49 (12.9)	12 (8.3)	37 (15.6)	
	Gastrointestinal		28 (7.3)	17 (11.8)	11 (4.6)	
	Cardiovascular		20 (5.2)	8 (5.6)	12 (5.1)	
	Endocrinological		19 (5.0)	5 (3.5)	14 (5.9)	
	Musculoskeletal		16 (4.2)	2 (1.4)	14 (5.9)	
	Trauma		16 (4.2)	4 (2.8)	12 (5.1)	
	Other		19 (5.0)	12 (8.3)	7 (3.0)	
Tube feeding, *n*			105 (27.6)	49 (34.0)	56 (23.6)	0.028
Central parenteral nutrition, *n*			44 (11.5)	18 (12.5)	26 (11.0)	0.741
Dementia, *n*			245 (64.3)	92 (63.9)	153 (64.6)	0.895
Charlson comorbidity index, n	Low		65 (17.1)	23 (16.0)	42 (17.7)	0.663
	Medium		165 (43.3)	63 (43.8)	102 (43.0)	
	High		106 (27.8)	44 (30.6)	62 (26.2)	
	Very high		45 (11.8)	14 (9.7)	31 (13.1)	
BI at the beginning			0 (0-5)	0 (0-5)	0 (0-10)	0.004
MNA-SF at the beginning, *n*		Normal	2 (0.5)	0 (0.0)	2 (0.8)	0.277
		At risk	94 (24.8)	30 (21.0)	64 (27.1)	
		Malnutrition	283 (74.7)	113 (79.0)	170 (72.0)	
TP, g/dl			6.4 (0.9)	6.3 (0.8)	6.4 (1.0)	0.143
ALB, d/dl			3.2 (0.7)	3.1 (0.6)	3.2 (0.7)	0.032
BUN, mg/dl			29 (17.9-44.7)	29.9 (18.5-47.2)	28.2 (17.7-43.1)	0.521
TLC, /µL			767 (460-1170)	798 (463-1168)	715 (461-1186)	0.818
Rehabilitation time, hour			8 (4-13)	9 (4-14.8)	7.5 (4.5-11.5)	0.051
Energy sufficiency in BEE, %			77.2 (35.2)	78.9 (33.7)	76.1 (36.1)	0.465

**Figure 2:**
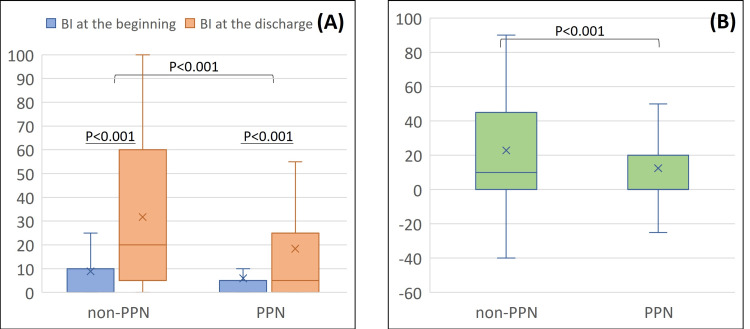
(A) Comparison of the Barthel index at the beginning of rehabilitation and at the discharge by PPN. (B) Difference between the beginning of rehabilitation and the discharge by PPN BI, Barthel index; PPN, peripheral parenteral nutrition

The primary outcome is displayed in Figure 2.

Both groups had a better BI at discharge compared to that at the beginning of rehabilitation; however, contrary to expectations, the improvement was significantly lower in the PPN group, both in the univariate and multivariable analyses (Table 2).

**Table 2:** Multivariate analysis on the difference in BIs between the beginning and the discharge by PPN or not BI, Barthel index; PPN, peripheral parenteral nutrition; CI, confidence interval, *adjusted covariates: age, tube feeding, central parenteral nutrition, dementia, albumin at the admission, Charlson comorbidity score, ICU or HUC stay, Barthel index at the beginning

	Difference in BI, median (IQR)	p-value	Unadjusted β coefficient (95% CI)	Multivariate ^*^adjusted β coefficient (95% CI)
Non-PPN (n=238)	10 (0-45)	p<0.01	0 (reference)	0 (reference)
PPN (n=144)	0 (0-20)		-10.4 (-16~-4.8)	-7.2 (-12.7~-1.8)

The rates of transfer for rehabilitation purposes and change in biochemical nutritional indices (total protein, albumin, and urea nitrogen concentration and total lymphocyte count) did not differ between the two groups. Similar results were obtained in the multivariable analysis (Table 3).

**Table 3:** Multivariate analysis in the secondary outcomes $1 adjusted covariates (age, tube feeding, central parenteral nutrition, dementia, albumin at admission, Charlson comorbidity score, ICU or HCU stay, Barthel index at the beginning), $2 adjusted covariates (age, tube feeding, central parenteral nutrition, Charlson comorbidity score, ICU or HCU stay, total protein at admission), $3 adjusted covariates (age, tube feeding, central parenteral nutrition, Charlson comorbidity score, ICU or HCU stay, albumin at admission), $4 adjusted covariates (age, tube feeding, central parenteral nutrition, Charlson comorbidity score, ICU or HCU stay, blood urea nitrogen at admission), $5 adjusted covariates (age, tube feeding, central parenteral nutrition, Charlson comorbidity score, ICU or HCU stay, total lymphocyte count at admission)

			p-value	Multivariate adjusted (95% CI)
Rehabilitation transfer odds raito (95% CI)		1.13 (0.733-1.753)	p=0.553	1.10 (0.683-1.780)^$1^
Difference in TP, mean (SD)	Non-PPN	-0.5 (0.60)	p=0.309	-0.0 (-0.18~0.16)^$2^
	PPN	-0.4 (0.94)		
Difference in ALB, mean (SD)	Non-PPN	-0.5 (0.60)	p=0.107	-0.0 (-0.09~0.09)^$3^
	PPN	-0.4 (0.60)		
Difference in BUN, median (IQR)	Non-PPN	-7.8 (-21.3~-0.1)	p=0.842	1.0 (-1.90~4.00)^$4^
	PPN	-9.3 (-21.9~-0.2)		
Difference in TLC, mean (SD)	Non-PPN	175.3 (930.36)	p=0.058	63.0 (-64.66~190.56)^$5^
	PPN	356.3 (682.75)		

**Figure 3:**
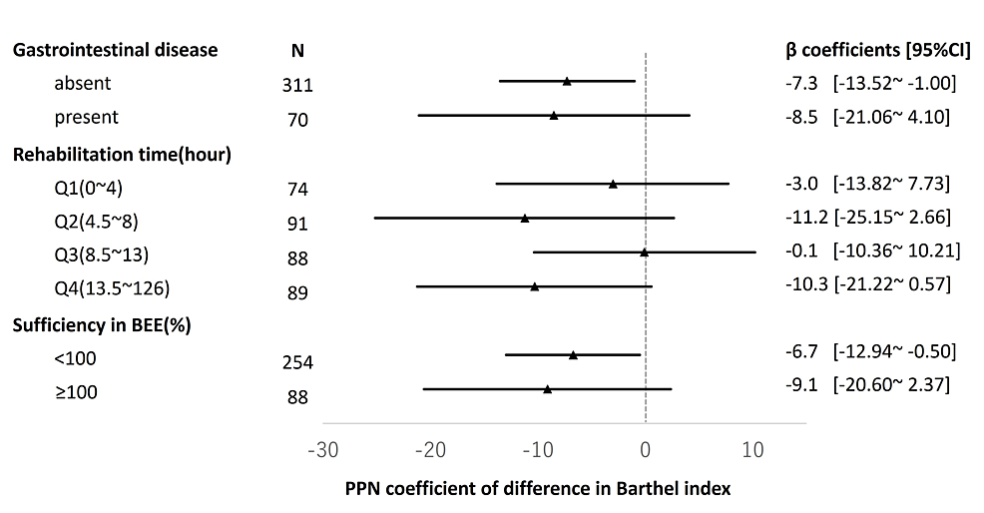
Stratified analysis by gastrointestinal disease, rehabilitation time, and sufficiency in basal energy expenditure Adjusted covariates: age, tube feeding, central parenteral nutrition, dementia, albumin at the admission, Charlson comorbidity score, ICU or HUC stay, and Barthel index at the beginning PPN, peripheral parenteral nutrition; BEE, basal energy expenditure

Upon the subgroup analysis, PPN remained an inhibitory factor for ADL recovery in all subgroups (Figure 3).

**Figure 4:**
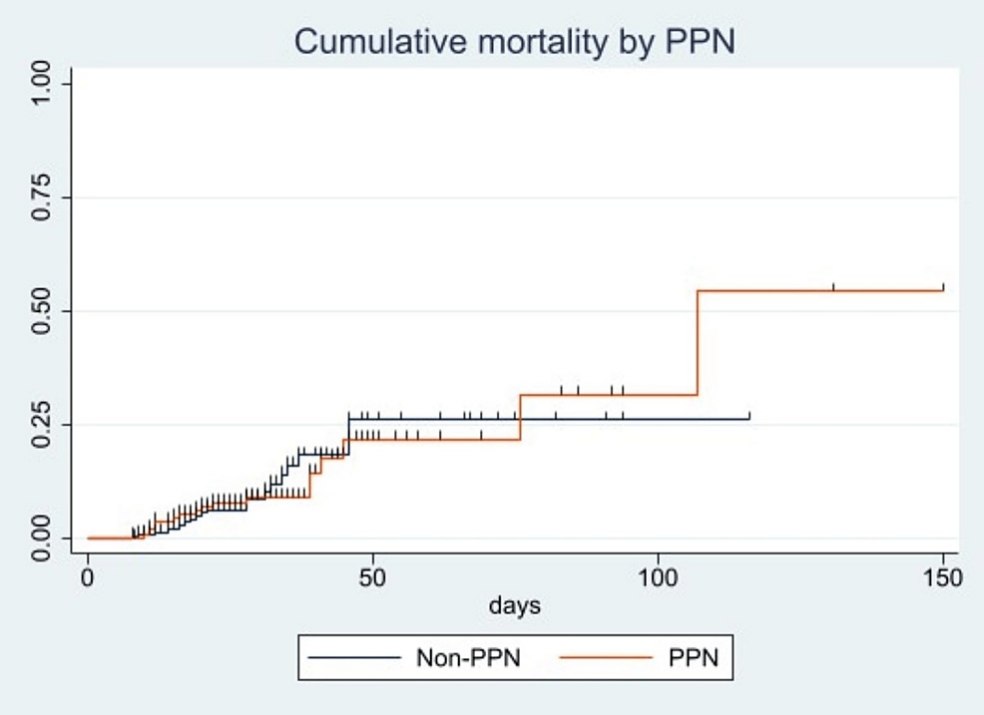
Cumulative mortality rates in the PPN and non-PPN groups

The cumulative mortality rates in the PPN and non-PPN groups did not significantly differ (Figure 4).

Moreover, the results of the sensitivity analysis were the same trend as that of the main analysis (Table 4).

**Table 4:** Sensitivity analysis including death cases BI, Barthel index; PPN, peripheral parenteral nutrition; CI, confidence interval; *adjusted covariates: age, tube feeding, central parenteral nutrition, dementia, albumin at the admission, Charlson comorbidity score, ICU or HUC stay, Barthel index at the beginning

	Multivariate^*^ adjusted β coefficient (95% CI)	
	Complete case analysis n=342	Sensitivity analysis n=379
Non-PPN	0 (reference)	0 (reference)
PPN	-7.2 (-12.7~-1.8)	-7.6 (-12.6~-2.6)

## Discussion

The purpose of this study was to determine whether PPN is useful for frail older adults who are unable to obtain a sufficient oral caloric intake. PPN did not promote the recovery of the BI; in fact, contrary to expectations, the recovery of BI was significantly better in the non-PPN group, even after adjusting for multiple confounding factors.

To the best of our knowledge, this is the first study in which the effect of PPN on frail older adults was examined during their hospital stay. A number of studies have demonstrated the usefulness of PPN administered for short periods during the perioperative period for gastrointestinal malignancies [21-23]. However, patients with surgical indications in those studies had a performance status of 0-1, which is better than that of almost all of our study participants.

Many frail older adults have sarcopenia due to malnutrition, and their physical function is easily compromised by acute illnesses and other factors. In a systematic review and meta-analysis, Ligthart-Melis et al. [7] examined the prevalence of frailty, sarcopenia, and malnutrition in adults aged 60 years and older. They discovered that the prevalence of frailty was 84%, with a strong association with malnutrition (odds ratio = 5.77). The proportion of patients with malnutrition was also high in our study of hospitalized, frail older patients (78.5% and 71.9% in the PPN and non-PPN groups, respectively). Milne et al. [24] reported that oral nutritional supplementation during short-term hospitalization of poorly nourished older patients reduced complications and mortality. Lærum-Onsager et al. [25] also reported that post-hospitalization nutritional interventions may reduce readmissions of older patients within 90 days. Nutritional interventions for frail older adults have proven beneficial in combination with other interventions, particularly exercise therapy, in several systematic reviews [10,26]. However, most of the studies under review did not involve patients hospitalized for acute illnesses, but rather those living in the community or in institutional care. Frail patients hospitalized for acute illnesses are often unable to obtain sufficient energy via oral intake, including oral nutritional supplements. In such patients, nutritional intervention with PPN is thought to be more beneficial than regular electrolyte infusions alone, as it may restore physical activity, which, in turn, would improve oral intake. However, the opposite was true in our study: the PPN group exhibited a poorer ADL recovery. Subgroup analysis revealed that, in the PPN group, ADL recovery was poor even in the group with an energy intake sufficiency of 100% or more compared to their basal energy expenditure, suggesting that factors other than malnutrition played a role in patients’ ADL recovery.

One factor may be the intensity of the inpatient rehabilitation exercises. Previous studies have demonstrated the benefits of nutritional interventions in combination with exercise therapy in frail older patients. Nutrition alone has not proven beneficial [6,27], and recovery of muscle strength is largely dependent on exercise intensity. Patients receiving PPN may not be able to exercise with sufficient intensity owing to the intravenous line. In addition, older patients often have poor cardiac and renal functionality, and PPN use, which increases fluid volume, may result in systemic edema and pleural effusion. Such systemic edema and pleural effusion may prevent patients from increasing their exercise intensity. In a retrospective analysis of older hospitalized patients with poor nutritional status, Hiramatsu et al. [28] examined the risk factors for unsuccessful nutrition support team intervention. Interestingly, PPN use was a risk factor for failure of NST intervention, with a hazard ratio of 1.80. Taken together with the results of this study, the use of PPNs in hospitalized olfrt patients with poor nutritional status may not always be beneficial. However, a recent systematic review revealed that no interventions had strong evidence backing their ability to prevent muscle weakness in hospitalized older patients [29]. Further studies are necessary to determine how to prevent muscle weakness during hospitalization in such patients, especially those with frailty.

Another possible factor in older patients’ ADL recovery may be overfeeding. Casaer et al. [30] compared the nutritional management of critically ill patients with enteral nutrition alone to that with enteral nutrition plus parenteral nutrition. They reported longer ICU stays, increased rates of new infections, and longer durations of mechanical ventilation and renal replacement therapy in the group that received parenteral nutrition in addition to enteral nutrition. They hypothesized that the suppression of autophagy owing to overfeeding causes delayed recovery from infection and organ failure. Although only approximately 30% of the critically ill patients in this study stayed in the ICU or HCU, many patients had infectious diseases, such as pneumonia, urinary tract infections, bacteremia, and sepsis, suggesting a possible influence of autophagy disorders.

Our study has several limitations. First, the decision to use PPN was left to the attending physician. Although the two groups did not differ in terms of nutritional status or CCI at admission, a selection bias might have been present; patients in the PPN group might have had a poorer prognosis because of the severity of the acute illness that precipitated admission or because of comorbidities that are not assessed with the CCI. Second, this study is an observational study, not an intervention study, so the dosage, timing, and duration of PPN are not standardized. The possibility remains that PPN can be effective with appropriate standardized dosing. Third, this was a retrospective study, so confounding factors, such as sarcopenia, which are not measured in routine practice, were not adjusted for. As sarcopenia affects ADL recovery and might have influenced the attending physician’s choice to use PPN, it might have been a confounding factor in this study, skewing results. Fourth, the only outcome measure of physical activity was ADLs. Previous interventional studies for frailty have included multiple outcomes, such as frailty indices, muscle strength, body composition, physical activity, the Short Physical Performance Battery, the Timed Up and Go Test, and walking speed. Owing to the retrospective nature of this study, these outcomes, which were not measured in the clinical setting, could not be used. Finally, this was a single-center retrospective study. Therefore, caution should be exercised when applying the results of this study to other institutions. A larger, multicenter, randomized, controlled trial is needed to verify our results.

## Conclusions

When frail patients are hospitalized for acute illness and their oral intake is inadequate, PPN may not have a beneficial effect on their nutritional status or ADL recovery. Because this is a retrospective observational study and many limitations exist, the usefulness of PPN could not be completely denied. Appropriate inpatient management strategies for frail patients who do not receive adequate oral nutrition requires further investigation.
